# Variations of Antioxidant Characteristics and Mineral Contents in Pulp and Peel of Different Apple (*Malus domestica* Borkh.) Cultivars from Pakistan

**DOI:** 10.3390/molecules17010390

**Published:** 2012-01-04

**Authors:** Maleeha Manzoor, Farooq Anwar, Nazamid Saari, Muhammad Ashraf

**Affiliations:** 1 Department of Chemistry and Biochemistry, University of Agriculture, Faisalabad-38040, Pakistan; 2 Department of Chemistry, University of Sargodha, Sargodha-40100, Pakistan; 3 Faculty of Food Science and Technology, Universiti Putra Malaysia, UPM-43400 Serdang, Selangor, Malaysia; 4 Department of Botany, University of Agriculture, Faisalabad-38040, Pakistan; 5 Department of Botany and Microbiology, King Saud University, Riyadh 11451, Saudi Arabia

**Keywords:** phenolic compounds, DPPH radical, antioxidant activity, *Malus domestica*, *Inter-cultivar variation*, mineral elements, fruit tissue

## Abstract

Variations of phenolics, antioxidant activity, and mineral contents in peel and pulp of five apple (*Malus domestica* Borkh.) cultivars from Pakistan, namely Red Delicious, Golden Delicious, Kashmiri Amri, Kala Kulu and Sky Spur were appraised. The mean extract yield of antioxidant components obtained with 80:20 methanol-water (v/v), was found to be 22.1 g/100 g for peel and 14.2 g/100 g for pulp on a dry weight basis. The amounts of total phenolics and total flavonoids in peel and pulp of different cultivars of apple ranged from 1,907.5–2,587.9 mg gallic acid equivalent/100 g DW and 1,214.3–1,816.4 mg catechin equivalent/100 g DW and 1,185.2–1,475.5 mg GAE/100 g DW and 711.8–999.3 mg CE/100 g DW, respectively. The inhibition of linoleic acid peroxidation and DPPH scavenging activity of the extracts varied from 71.7–84.9 and 66.6–80.8% in peel, and 43.9–52.8 and 42.9–51.1% in pulp, respectively. Reducing power of the tested fruit part extracts at concentration 12.5 mg/mL ranged from 2.54–2.89 and 1.37–1.73, respectively. With regard to minerals analysis, both fruit parts showed the amount of K to be the highest, followed by Mg, Ca, Fe, Na and Zn. The results revealed that peel of the tested apple cultivars in this study had superior antioxidant capacity and mineral concentration than the pulp, indicating significant variations between the parts tested. Thus, consumption of apple fruits along with peel might be recommended to gaining better nutritive benefits.

## 1. Introduction

It is widely accepted that dietary intake of selected fruits, especially those containing functional bioactives such as phenolic acids, tannins, flavonoids, and vitamins and essential minerals, is linked to reduced prevalence of several chronic diseases [1,2,3]. Apple (*Malus domestica* Borkh.), a member of the family *Rosaceae*, is one of the most frequently consumed fruits in many regions across the World. Apple fruit has been identified as an excellent potential source of carbohydrates, minerals, dietary fiber and antioxidant phenolics [3,4,5]. Recent studies reveal that consumption of apple fruit and apple juice can provide antiproliferative, anticarcinogenic, and anti-inflammatory health benefits and is strongly associated with lower incidence of lung cancer, viral diseases and cardiovascular disorders [1,3,6,7,8]. Apple peel, being a rich source of phenolics and dietary fiber, is also valued for its medicinal health functions [5,6].

The composition and distribution of nutrients and high-value components such as phenolics mainly depends upon genotypes, fruit tissue and the maturity levels of fruits and to a smaller extent on environmental aspects [9,10,11,12]. Unpeeled fruits possess higher contents of bioactive compounds as compared to peeled ones [8]. The fruit peels, although medicinally important, are often discarded as an agro-waste and have not yet received attention with regard to their utilization for value-addition rather than being discarded. This might be due to the lack of commercial applications and consumer’s unawareness of the benefits of consuming fruit peels. Interestingly, the seed and peel fractions have been reported to have higher antioxidant activity compared to the pulp fraction. Gorinstein *et al*. [13] reported that the mineral and total phenolic contents in persimmon and apple peel were significantly higher compared to those in pulp. Similarly, Loentowicz *et al*. [8] reported that apple and pear peel extracts exhibited better potential as natural antioxidant supplements than pulp extract. 

Pakistan is blessed with a wide range of agro-climatic conditions, which allow the production of both tropical and temperate fruits [14]. The most suitable soil and climatic conditions for apple cultivation prevail in the northern hilly areas of Punjab, Khyber Pukhtonkha and Baluchistan. Apple is the fourth major fruit of Pakistan after citrus, mango and banana and is widely consumed. Various cultivars of apple being grown in Pakistan include Mashaday, Kashmiri Amri, Red Delicious, Sky Spur, Kala Kulu, Golden Delicious, *etc.* [15,16,17]. The total area under apple cultivation was estimated to be 112.6-thousand ha, while total production was as high as 348.3 thousand tons [18]. Several studies have been carried out on the phenolic profile and antioxidant potential of different cultivars and parts of apple fruit from various regions [1,4,5,9,13,19,20,21]. However, to the best of our knowledge, no reports on the antioxidant attribute and mineral composition of different parts of apple cultivars commonly grown in Pakistan is available in the literature. Thus, this study was undertaken to quantify and compare the phenolic components, antioxidant activity and mineral contents in pulp and peel of five cultivars widely cultivated in Pakistan. 

## 2. Results and Discussion

### 2.1. Extract Yields, Total Phenolic and Total Flavonoid Contents

The peel and pulp of fruits of the studied cultivars of apple yielded promising amounts of extractable matter (dry weight basis) with aqueous methanol. The mean extraction yield of antioxidant components was found to be 22.1 g/100 g for peel and 14.2 g/100 g for pulp. Relatively a higher extraction yield was obtained for peel than the pulp samples. The significant differences in the extraction yield between peel and pulp of same fruits might be ascribed to the varying availability of extractable components, resulting from the varied chemical composition of the different tissues used. Li *et al*. [22] also reported that percent yield of pomegranate peel (31.5%) extract was higher compared to that of pulp (14.5%) extract.

**Table 1:** Total phenolics and total flavonoids contents of peel and pulp extracts of different cultivars of apple (*Malus domestica* Borkh.) fruit.

Variety	TPC (mg gallic acid equivalent/100 g dry weight)		TFC (mg catechin equivalent/100 g dry weight)	
	Peel	Pulp	Peel	Pulp
Golden Delicious	2102.4 ± 44.1 ^b^	1298.2 ± 26.8 ^a^	1501.5 ± 31.3 ^a^	816.3 ± 16.9 ^a^
Red Delicious	2587.9 ± 50.6 ^a^	1475.5 ± 29.9 ^a^	1816.4 ± 36.1 ^a^	930.2 ± 19.9 ^a^
Kashmiri Amri	2097.1 ± 43.4 ^b^	1185.2 ± 24.7 ^ab^	1398.4 ± 27.9 ^ab^	789.3 ± 15.8 ^a^
Kala Kulu	2274.8 ± 49.4 ^b^	1388.4 ± 26.1 ^a^	1694.6 ± 37.2 ^a^	999.3 ± 17.7 ^a^
Sky Spur	1907.5 ± 38.9 ^b^	1201.2 ± 24.1 ^a^	1214.3 ± 24.1 ^b^	711.8 ± 21.2 ^ab^
**Mean**	2193.9 ± 43.9 _a_	1309.7 ± 27.4 _b_	1525.0 ± 31.9 _a_	849.4 ± 17.9 _b_

Data are mean ± SD (*n* =3 × 3, *P* < 0.05). Different superscript letters within the same column indicate significant differences (*P* < 0.05) among cultivars. Different subscript letters within the Mean’s row indicate significant differences (*P* < 0.05) between peel and pulp.

We analyzed TPC and TFC of the apple fruit extracts on a dry weight (DW) basis using the spectrophotometric method. Plant phenolics, with more than 8,000 chemical structures at present identified, are known to act as an effective free radical scavengers and antioxidants [22,23]. The most important natural phenolics are flavonoids because of their broad spectrum biochemical and biological activities. Thus, the true antioxidant potential is often more accurately revealed by expressing antioxidant activity in terms of total phenolics and flavonoids [23,24].

The contents of total phenolics, calculated as gallic acid equivalent (GAE), of 80% methanolic extracts of apple peel and pulp are presented in Table 1. Total phenolic contents ranged from 1,907.5–2,587.9 mg GAE/100 g in the peel extracts and 1,185.2–1,475.5 mg GAE/100 g in the pulp extracts. Of the cultivars tested, Red Delicious and Kala Kulu peel extracts exhibited significantly higher contents of total phenolics, 2,587.9 and 2,274.8 mg/100 g, respectively, followed by Golden Delicious (2,102.4 mg/100 g) and Kashmiri Amri (2,097.1 mg/100 g). The lowest (1,907.5 mg/100 g) content of total phenolics was observed for Sky Spur. In case of pulp extracts, highest (1,475.5 mg/100 g) amount of total phenolics (TP) was exhibited by Red Delicious apple and the lowest (1,185.2 mg/100 g) by Kashmiri Amri. The amount of TP in the pulp extracts of Golden Delicious and Sky Spur apple were 1,298.2 and 1,201.2 mg/100 g, respectively.

The amount of total soluble flavonoids, expressed as catechin equivalent (CE), among peel of fruits of five apple cultivars ranged from 1,214.3 to 1,816.4 mg CE/100 g of extract (Table 1). The peel extracts of Red Delicious apple exhibited the highest (1,816.4 mg/100 g) flavonoid contents followed by Kala Kulu (1,694.6 mg/100 g). These contents were higher than those for the peel extracts from Golden Delicious (1,501.5 mg/100 g), Kashmir Amri (1,398.4 mg/100 g) and Sky Spur (1,214.3 mg CE/100 g). For pulp extracts, the amounts of total flavonoids (TF) varied from 711.8 mg CE/100 g (Sky Spur) to 999.3 mg/100 g (Kala Kulu). 

Overall, there was no considerable variation in the amounts of TP and TF among the five apple cultivars, however these levels differed significantly between the two parts (peel and pulp) tested. As expected, the peel of all the cultivars analyzed had significantly higher concentrations of TP and TF than those determined for pulp. The results of our present investigation are in agreement with those reported by Vieira *et al*. [19], who found that peel extract of different varieties of apple fruit contained greater amount of phenolics, anthocyanins and flavonoids than pulp extract. In agreement to our present data, Abrosca *et al*. [1] reported that apple peel extracts have higher amount of phenolics and flavonoids than pulp extracts. Likewise, in another study by Drogoudi *et al*. [20], apple peel extracts exhibited 1.5 to 9.2 times greater total antioxidant activity and 1.2 to 3.3 times higher contents of total phenolics than those of flesh (pulp) extracts. 

In order to assess the dietary impact of consumption of apple fruit on the intake of antioxidant phenolics, their supplies on the basis of fruit serving were calculated. The calculation was based on fresh fruit serving of 100 g which comprised of 80 g pulp + 15 g skin + 5 g ovary with seeds. On the basis of this calculation, TP and TF contents were ranged from 38.1–60.2 and 26.2–40.2 mg/serving, respectively. The highest contribution of TP and TF was provided by Red Delicious (60.2 and 40.2 mg/serving) followed by Kala Kulu (53.9 and 34.4 mg/serving) and Golden Delicious (45.2 and 30.8 mg/serving) whereas, lowest by Kashmiri Amri (38.7 and 26.2 mg/serving) and Sky Spur (38.1 and 28.8 mg/serving), respectively. 

In general, it is observed that apple peel possess higher contents of phenolics and flavonoids compared to the pulp, because of the nature and distribution of phenolics and flavonoids varies within different parts of a same fruit. The pulp of apple fruit contains catechin, phloretin glycoside, procyanidins, caffeic acid; the peel possesses all of these compounds and has additional flavonoids such as anthocyanins, quercetin glycosides and cyanidin glycoside, not present in the pulp [25]. The presence of higher amounts of total phenolics and flavonoids in peel, compared with pulp, in the present analysis of apple fruits can also be justified by the findings of other researchers [1,8,9,20].

### 2.2. Reducing Power of Apple Extract

Measurement of reducing power also defines an important aspect of antioxidant activity of plant extracts. In this assay, the presence of reducing agent in the analyte sample results in the reduction of the ferric/ferricyanide complex to its ferrous (Fe^+2^) form. The amount of Fe^2+^ is then quantitatively monitored by measuring the intensity of Perl’s Prussian blue color complex at 700 nm. Higher absorbance value indicates higher reducing power and thus higher antioxidant activity [24].

Table 2 presents the reductive capability of peel and pulp extracts of different apple cultivars. In the reducing power assay, (extract concentrations used were: 2.5, 5.0, 7.5, 10.0 and 12.5 mg/mL), almost a concentration-dependent increase in the absorbance of the samples tested was recorded (data not shown).

**Table 2:** Reducing power (absorbance values at 700 nm) of peel and pulp extracts (at concentration 12.5 mg/mL) from different cultivars of apple (*Malus domestica* Borkh.) fruit.

Variety	Peel	Pulp
Golden Delicious	2.66 ± 0.05 _a_^a^	1.60 ± 0.04 _b_^a^
Red Delicious	2.89 ± 0.04 _a_^a^	1.73 ± 0.04 _b_^a^
Kashmiri Amri	2.60 ± 0.06 _a_^a^	1.37 ± 0.02 _b_^a^
Kala Kulu	2.69 ± 0.07 _a_^a^	1.69 ± 0.03 _b_^a^
Sky Spur	2.54 ± 0.06 _a_^a^	1.49 ± 0.03 _b_^a^

Data are mean ± SD (*n* = 3 × 3, *P* < 0.05); Different superscript letters within the same column indicate significant differences (*P* < 0.05) among cultivars; Different subscript letters within the same row indicate significant differences (*P* < 0.05) between peel and pulp.

The reductive capability of apple fruit as determined in the present analysis was comparable to those reported in longan fruit extract [26]. Analysis of variance (ANOVA) indicated that the reductive capability of apple peel extracts was significantly higher than pulp extracts. This might be due to the fact that reductants/phenolics compounds are present in higher concentrations in the outer tissues (peel and sub-epidermal tissue) of the fruits than in the inner tissues (mesocarp and pulp) [1,19,25]. There was no significant cultivar effect on the reducing power of the peel and pulp.

### 2.3. DPPH Radical Scavenging Activity

The characteristic feature of antioxidants to scavenge DPPH free radical is well accepted and is therefore most often selected as a reliable tool to evaluate the free radical scavenging capacity of different plant extracts. Interestingly, the DPPH radical assay is incredibly sensitive towards active ingredients even at lower concentrations. Another beneficial aspect is that this test is time saving and can be used to analyze a batch of samples in a shorter time. This procedure has been often used for the assessment of free radical scavenging ability of plant-based antioxidant extracts [27]. It is generally predicted that DPPH radical scavenging activity, and with it antioxidant activity, is strongly affected by the amount of phenolic compounds as well as the degree of hydroxylation of the phenolic compounds [28,29].

**Figure 1:**
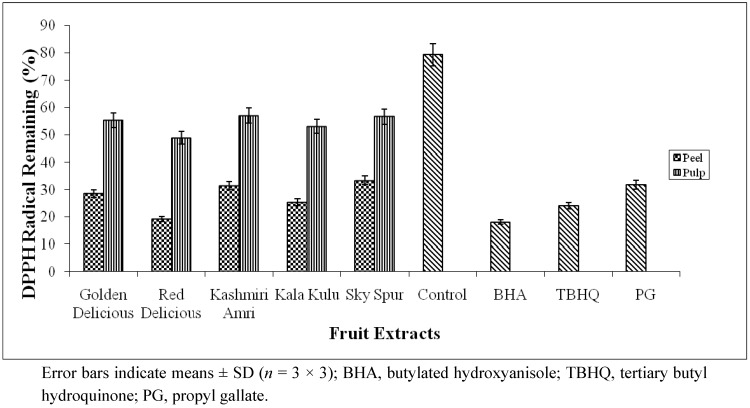
DPPH radical scavenging activity of 80% methanolic extracts of peel and pulp from different cultivars of apple (*Malus domestica* Borkh.) fruit.

The DPPH radical scavenging activity of peel and pulp extracts was tested and compared with BHA, TBHQ and PG (Figure 1). The absorbance in this assay was recorded at different intervals (starting with 1 to 12 min) from the beginning of the reaction. Highest variation in scavenging activity, observed at 5^th^ min of the reaction, was used for calculation purposes. The scavenging activity of positive control, BHA was found to be higher than those of the peel and pulp extracts. Other positive controls employed namely TBHQ and PG exhibited comparable scavenging activity with peel extracts, but showed higher potential than the pulp extracts.

As depicted, the peel extracts derived from all apple cultivars exhibited appreciably higher scavenging activity, ranging from 66.6–80.8%, compared to pulp extracts, 42.9–51.1% (Figure 1). The peel extract from Red Delicious showed the highest (80.8%) scavenging activity, whereas lowest (66.6%) for Sky Spur. In case of pulp extracts, the highest (51.1%) and the lowest (42.9%) scavenging capacity was recorded for Red Delicious and Kashmiri Amri, respectively. In close agreement to our present data, in a previous study by Abrosca *et al*. [1], apple peel and pulp extracts (at 1.0 mg/mL) showed 78% and 55% DPPH radical scavenging capacity, respectively.

No significant variation of radical scavenging capacity, was observed among the tested apple cultivars, however, the difference between peel and pulp parts of the fruits was significant. High radical scavenging activity of peel extract compared with that of pulp extract might be linked to the presence of greater amount of phenolic compounds, flavonols and anthocyanins in the former. It has been reported that free radical scavenging activity of plant based food (fruits and vegetables) extracts is mainly due to phenolic compounds, which are mostly distributed in the epidermal tissue [1,30]. It is widely accepted that antioxidant activity of plant-derived extracts is largely due to the ability of intrinsic phenolics to donate hydrogen atoms or electrons to capture the free radicals [31].

### 2.4. Antioxidant Activity of Apple Peel and Pulp Extracts in Linoleic Acid Peroxidation System

**Figure 2:**
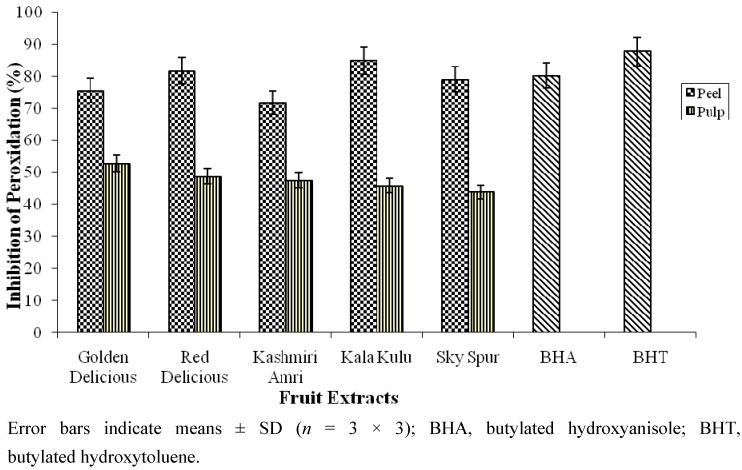
Antioxidant activity of 80% methanolic extracts of peel and pulp from different cultivars of apple (*Malus domestica* Borkh.) fruit.

The inhibition of linoleic acid peroxidation was also used to appraise the antioxidant activity of apple fruit extracts. Both apple fruit parts (peel and pulp) exhibited appreciable inhibition of linoleic peroxidation as shown in Figure 2. The inhibition of peroxidation ranged from 71.7 to 84.9% for the peel extract, whereas 43.9 to 52.8% for the pulp extract. The peel of Kala Kula and Kashmiri Amri exhibited the highest (84.9%) and lowest (71.7%) inhibition of peroxidation, whereas; in case of pulp extract Golden Delicious and Sky Spur offered the highest (52.8%) and lowest (43.9%) inhibition of peroxidation, respectively.

A significant difference in percent inhibition of peroxidation was observed between peel and pulp parts of the five cultivars of apple fruit. However, variation of these data among different cultivars was found to be non-significant. Considerable inhibition of linoleic acid peroxidation by peel and pulp extracts tested might be attributed to the presence of phenolics, anthocyanins, epicatechin and flavone-3-ol in these fruits [25].

### 2.5. Minerals

Minerals play a key role in various physiological functions of the body, especially in the building and regulation processes [32]. Apples are considered as a good source of dietary minerals. The results as given in Table 3 of the main mineral and trace elements of the tested apple cultivars showed that all the samples analyzed contained higher amount of potassium (K) followed by calcium (Ca), magnesium (Mg) and sodium (Na). The contents of K and Ca in apple peel and pulp ranged from 695.3–980.9 and 443.6–790.1 mg/100 g DW and 35.6–72.1 and 19.8–48.9 mg/100 g DW, respectively. The peel and pulp of Golden Delicious exhibited the highest contents of K (980.9 and 790.1 mg/100 g), whereas, lowest (695.3 and 443.6 mg/100 g) in Sky Spur, respectively. The concentration of Ca was highest in peel (72.1 mg/100 g) and pulp (48.9 mg/100 g) of Red Delicious while lowest in peel (35.6 mg/100 g) and pulp (19.8 mg/100 g) of Sky Spur.

Potassium is an essential element for normal body growth and maintenance. It also plays a significant role in the osmoregulation of cells and tissues. Potassium deficiency may cause a variety of disorders such as muscular cramps, twitching, and irregular heart beat and kidney failure [32,33]. Calcium is an important constituent of bones and teeth and it is actively involved in the regulation of nerve and muscle functions [34].

The contents of Mg and Na ranged from 35.7–65.9 and 2.9–7.3 mg/100 g for peel while 15.6–34.8 and 5.3–10.8 mg/100 g for pulp, respectively. In case of peel, Red Delicious and Kashmiri Amri exhibited the highest (65.9 mg/100 g) and lowest (35.7 mg/100 g) values, whereas for pulp, Red Delicious and Sky Spur showed the maximum (34.8 mg/100 g) and minimum (15.6 mg/100 g) content of Mg, respectively. Interestingly, the peel and pulp of Golden Delicious had 7.3 and 10.8 mg/100 g and Kashmiri Amri 2.9 and 5.3 mg/100 g of Na, respectively.

With regard to the amounts of two essential trace elements, the content of Fe in peel (1.2–2.4 mg/100 g) and pulp (0.8–2.1 mg/100 g) of the tested apple cultivars was higher compared to Zn, 0.4–1.2 mg/100 g in peel and 0.2–0.9 mg/100 g in pulp. The highest content of both of these trace elements was found in Golden Delicious and the lowest in Sky Spur. Being good sources of these two essential elements that are in short supply in the normal human diet, the tested fruits, especially Golden Delicious type apples, can be explored as a potential source of Zn and Fe [35].

The calculation of K, Ca, Mg and Na on a fresh apple serving portion basis was made similarly as for TP and TF contents. The amount of K, Ca, Mg and Na in Kala Kulu was found to be highest (160.7, 11.8, 8.2 and 2.8 mg/serving), followed by Golden Delicious (143.9, 13.1, 5.72 and 1.6 mg/serving), Red Delicious (109.1, 14.1, 6.3 and 1.1 mg/serving), Sky Spur (90.11, 5.9, 3.9 and 1.7 mg/serving) and Kashmiri Amri (89.9, 6.24, 3.69 and 0.79 mg/serving), respectively. The contents of essential trace elements, namely Fe and Zn ranged from 0.11–0.35 and 0.04–0.22 mg/serving, with the highest value (0.35 and 0.22 mg/serving) exhibited by Kala Kulu while lowest was seen for Sky Spur (0.11 and 0.04 mg/serving).

Henriquez *et al*. [21] analyzed important minerals in peel and pulp of five different varieties of apple fruit on a wet basis, and found the following concentrations: 115.1–165.1 and 68.0–107.7 (mg/100 g of K), 9.8–17.9 and 4–4.7 (mg/100 g of Ca), 21.9–25.5 and 6.3–7.3 (mg/kg of Mg), 7.0–13.0 and 1.6-6.8 (mg/kg of Na), 0.84–0.99 and 0.24–0.55 (mg/kg of Fe), 0.05–0.13 and 0.00–0.03 (mg/kg of Zn), respectively.

The present minerals analysis results were in agreement with those of Nour *et al*. [36] who reported that potassium (K) was the most abundant nutrient in fruits of different apple varieties, followed by magnesium (Mg), calcium (Ca), sodium (Na), and iron (Fe). The content of K and Mg as determined in our present investigation was found to lower than those reported by Ekholm *et al*. [37] for apple peel (1,250 and 70 mg/100 g DW) and peeled *Malus* sp. (960 and 50 mg/100 g DW), respectively, while the amount of Ca in both the apple peel (50 mg/100 g DW) and peeled *Malus* sp. (40 mg/100 g DW) was comparable with our present data. Several factors, e.g., variety, state of ripeness, soil type, soil condition, and irrigation regime may cause variation in the mineral and trace elemental contents in different types of fruits as well as within different parts of the same fruit [38].

**Table 3:** Mineral content in peel and pulp tissues of different cultivars of apple (*Malus domestica* Borkh.) fruit.

Variety	Part used	Mineral Contents (mg/100 g dry wt)		
		K	Ca	Mg
**Golden Delicious**	Peel	980.9 ± 21.4 ^a^	61.2 ± 1.32 ^a^	57.5 ± 1.18 ^a^
	Pulp	790.1 ± 17.9 ^bc^	36.7 ± 0.89 ^b^	23.9 ± 0.49 ^b^
**Red Delicious**	Peel	909.7 ± 17.3 ^b^	72.1 ±1.47 ^a^	65.9 ± 1.29 ^a^
	Pulp	693.8 ± 13.9 ^c^	48.9 ± 0.99 ^b^	34.8 ± 0.73 ^c^
**Kashmir Amri**	Peel	724.9 ± 15.5 ^c^	44.7 ± 0.92 ^b^	35.7 ± 0.69 ^c^
	Pulp	490.1 ± 11.2 ^d^	22.9 ± 0.67 ^d^	18.5 ± 0.37 ^bd^
**Kala Kulu**	Peel	833.8 ± 17.7 ^b^	52.2 ± 1.12 ^ab^	55.8 ± 1.21 ^ab^
	Pulp	550.7 ± 12.9 ^d^	30.5 ± 0.69 ^bc^	24.9 ± 0.49 ^d^
**Sky Spur**	Peel	695.3 ± 15.1 ^c^	35.6 ± 0.78 ^bc^	41.9 ± 0.87 ^c^
	Pulp	443.6 ± 11.7 ^de^	19.8 ± 0.41 ^d^	15.6 ± 0.35 ^d^
		Na	Fe	Zn
**Golden Delicious**	Peel	7.3 ± 0.21 ^b^	2.4 ± 0.04 ^a^	1.2 ± 0.04 ^a^
	Pulp	10.8 ± 0.29 ^a^	2.1 ± 0.02 ^a^	0.9 ± 0.01 ^a^
**Red Delicious**	Peel	4.7 ± 0.18 ^c^	1.8 ± 0.04 ^ab^	0.9 ± 0.06 ^a^
	Pulp	9.1 ± 0.17 ^a^	1.4 ± 0.05 ^b^	0.7 ± 0.03 ^b^
**Kashmir Amri**	Peel	2.9 ± 0.54 ^cd^	1.6 ± 0.05 ^b^	0.8 ± 0.05 ^ab^
	Pulp	5.3 ± 0.16 ^c^	1.1 ± 0.02 ^c^	0.5 ± 0.03 ^b^
**Kala Kulu**	Peel	5.9 ± 0.13 ^c^	2.2 ± 0.04 ^a^	1.0 ± 0.04 ^a^
	Pulp	8.2 ± 0.17 ^ab^	1.7 ± 0.03 ^b^	0.8 ± 0.03 ^ab^
**Sky Spur**	Peel	7.0 ± 0.11 ^b^	1.2 ± 0.02 ^bc^	0.4 ± 0.02 ^bc^
	Pulp	10.2 ± 0.09 ^a^	0.8 ± 0.13 ^c^	0.2 ± 0.03 ^c^

Data are mean (*n* = 3) SD ± (*n* = 3, *P* < 0.05); Different alphabets in superscript within the same column indicate significant differences (*P* < 0.05) between peel and pulp.

### 2.6. Correlations among the Results Obtained from Different Antioxidant Assays

As far as statistical evaluation of the results of different antioxidant assays is concerned, a good but varying correlation between TPC and TFC was observed for peel (*r* = 0.952 ***) and pulp (*r* = 0.846 **) extracts of apple fruit, as shown in Table 4. Lin and Tang [39] reported a strong correlation between total phenolics and flavonoids contents in selected fruits and vegetables. In a previous study by Vieira *et al*. [19], TPC and TFC were found to be strongly associated with total antioxidant activity in different parts (flesh, whole fruit and peel) of three cultivars of apple. These data reveal that phenolic compounds contribute significantly towards the antioxidant activity. Both the phenolic acids and flavonoids, being an important plant secondary metabolite, have been commonly recognized for their potent antioxidant and reductive capacities. Antioxidant activity of these compounds is mainly attributed to their redox potential [9]. Taking into account the examined parts and cultivars of fruits, in the present study, the extent of differences in phenolics and flavonoids among the apple cultivars tested was not so significant compared to that observed between the peel and pulp. In the present investigation, peel of different cultivars of apple fruit accounted for 1.5–2.0 fold higher antioxidant activity compared to the pulp extracts. Among the different cultivars tested Red Delicious contained highest amount of TP with its highest reduction potential. The correlation between TPC and reducing power of the peel and pulp extracts was highly significant (*r* = 0.976 *** and 0.915 ***) as presented in Table 4. 

**Table 4:** Comparison of results from different antioxidant assays as represented by correlation coefficient (*r*).

**Peel**	**Variable**	**TPC**	**TFC**	**%Inhibition**	**DPPH**	**Reducing power**
	**TPC**	1				
	**TFC**	0.952 ***	1			
	**%Inhibition**	0.480 ^ns^	0.561 ^ns^	1		
	**DPPH**	0.981 ***	0.965 ***	0.583 ^ns^	1	
	**Reducing power**	0.976 ***	0.912 ***	0.435 ^ns^	0.978 ***	1
**Pulp**	**TPC**	1				
	**TFC**	0.846 **	1			
	**%Inhibition**	0.245 ^ns^	0.116 ^ns^	1		
	**DPPH**	0.969 ***	0.744 *	0.178 ^ns^	1	
	**Reducing power**	0.915 ***	0.690 *	0.228 ^ns^	0.845 **	1

ns: non significant; *: level of significance.

In case of apple peel extract, a very good correlation between TFC and reducing power (*r* = 0.912 ***) and DDPH assay (*r* = 0.965 ***) was observed, however, this correlation was quite weak (*r* = 0.690 * and 0.744 *) in the case of the pulp extract. A highly positive correlation between TFC and reducing power for peel extract might be attributed to the fact that in peels quercetin glycosides, flavonols and anthocyanins, due to their high concentration, might have exhibited higher redox potential and radical scavenging activity compared to that of the pulp.

Likewise, a positive correlation between TPC and scavenging DPPH radical ability of apple peel and pulp extracts (*r* = 0.981 *** and 0.969 ***) was also established. The correlation between these two assays implied that the compounds liable for free radical scavenging activity might be mainly the phenolics. These phenolic compounds act as free radical scavengers, chain breakers, and electron donors. Previously, a similar type of high correlation between TPC and DPPH scavenging ability has been reported for apple and pear fruits peel and pulp extracts [8]. In agreement with our present data, Abrosca *et al*. [1] also found a good correlation between total phenolics contents and DPPH free radical scavenging ability of different apple cultivars. According to Wijngaard *et al*. [40], a significant correlation (*r* = 0.72) was observed between the results of total phenols and DPPH radical scavenging capacity of different fruits and vegetable byproducts, including apple fruit.

On the other hand, no significant correlation between the results of percent inhibition and reducing power assay for peel and pulp was recorded in the present experiments, which might be attributed to the fact that there may be some antioxidative compounds that exhibited their antioxidant activity not only by donating hydrogen but also by scavenging oxygen. Consequently, plant extracts with such antioxidant components might display higher degree of percent inhibition as compared to their corresponding reducing power and thus can offer no significant correlation. Furthermore, as expected, no significant correlation was observed between percent inhibition and TFC data of peel and pulp extracts which might be due to the reason that not only the flavonoids but several other compounds such as vitamins, anthocyanins, carotenoids *etc.* also contribute to antioxidant activity in terms of measurement of percent inhibition of peroxidation [41]. 

Similarly, no significant correlation between TPC and percent inhibition for peel and pulp extract was observed in the present analysis which could be explained on the basis that not only the phenolics but some other compounds such as tocopherols and carotenoids *etc.* also donate antioxidant activity in terms of measurement of percent inhibition of peroxidation [41]. Likewise our present trends, total phenolics and percent inhibition of linoleic peroxidation of different stored apple fruits showed a weak correlation [42]. The present variation in correlation coefficient among different antioxidant assays indicates that a single assay is not sufficient to fully appraise the antioxidant attributes of a specific plant matrix [43,44]. Besides, synergistic effects among different endogenous compounds are another factor which may also contribute to enhancing the total antioxidant capacity of plant extracts [45].

## 3. Experimental

### 3.1. Samples

Fresh fruits of five different apple cultivars, namely Golden Delicious, Red Delicious, Kashmiri Amri, Kala Kulu and Sky Spur, were collected from the vicinity of Swat city, Khyber Pukhtonkha, Pakistan and University of Azad Jammu & Kashmir, Rawalakot campus, Rawalakot, Azad Kashmir, Pakistan. Three different samples of each cultivar were randomly harvested. To ensure that the cultivars were the same, the fruits were further identified and authenticated by the Department of Horticulture, University of Agriculture, Faisalabad, Pakistan. 

### 3.2. Chemicals and Reagents

Linoleic acid, 2,2-diphenyl-1-picrylhydrazyl (DPPH) radical, Folin-Ciocalteu reagent, butylated hydroxyanisole (BHA) butylated hydroxytoluene (BHT), tertiary butyl hydroquinone (TBHQ), propyl gallate (PG) and gallic acid were purchased from Sigma Chemical Co. (St. Louis, MO, USA). All other chemicals and reagents (analytical grade) used were from Merck (Darmstadt, Germany) or Sigma Aldrich (Buchs, Switzerland), unless stated otherwise.

### 3.3. Sample Preparation

After washing with tap water, the fruits were peeled off with the aid of a steel knife. The seed were removed from the pulp manually and the pulp and peel recovered were sliced into approx. 1 × 1 cm slices.

### 3.4. Dry Matter Determination

In view of the varying levels of moisture among apple fruit varieties, we made all calculations on dry matter basis. For dry matter determination, AOAC procedure (method 925.10) was followed [46]. Briefly, known weight (5.0 g) of the sample was subjected to an electric-oven (Memmert, Germany) drying using 105 °C temperature, until constant weight achieved.

### 3.5. Antioxidant Activity of Fruits

#### 3.5.1. Extraction

Homogenized fruit sample (each 20 g) was extracted with aqueous methanol (200 mL, methanol-water, 80:20, v/v) at room temperature for 8 hours using an orbital shaker (Gallenkamp, UK). The residue was separated from the extract by filtering through Whatman filter paper No. 1 and re-extracted twice with fresh solvent. The three recovered extracts were pooled and then excess of the solvent was distilled off in a vacuum rotary evaporator (EYELA, Tokyo, Japan) at 45 °C. The semi-solid crude concentrated extracts obtained were stored at 4 °C, until used for further experimentation. 

#### 3.5.2. Determination of Extract Yield

The yield of extract (extractable components) expressed on dry weight basis of peel and pulp was calculated from the following equation:

Yield (g/100 g) = (W_1_ × 100)/W_2_

where W_1_ is the weight of the extract residue obtained after solvent removal and W_2_ is the weight of peel or pulp taken.

#### 3.5.3. Determination of Total Phenolics Content (TPC)

A calorimetric technique, based on Folin-Ciocalteu reagent, was employed to determine the amount of total phenolics [47]. The procedure involved the mixing of crude extract (50 mg) with Folin-Ciocalteu reagent (0.5 mL) and deionized water (7.5 mL). After allowing the mixture to stand for ten minutes at ambient (room) temperature, 20% aqueous sodium carbonate (w/v, 1.5 mL) was added. The mixture was incubated at 40 °C in a water bath for 20 min, followed by cooling using an ice bath. The absorbance of the final mixture was recorded at 755 nm (Spectrophotometer U-2001, Hitachi Instruments Inc., Tokyo, Japan). For calculation of TP amount, standard gallic acid calibration curve prepared by running solutions in the concentration range of 10–200 ppm (R^2^ = 0.9980) was used. The results were expressed as gallic acid equivalents (GAE) mg/100 g of dry matter. 

#### 3.5.4. Determination of Total Flavonoid Contents (TFC)

Amounts of TF were estimated calorimetrically. A previously described method [48] was used wherein fruit extract (1 mL containing 0.1 mg/mL dry matter) was mixed with water (4 mL) in a 10 mL volumetric flask. At the start, aqueous 5% NaNO_2_ (3 mL) was added to the volumetric flask then at 5 min, 10% AlCl_3_ (0.3 mL) and at 6 min, 1.0 *M* NaOH (2 mL) were added sequentially. Finally, the volume was made up to 10 mL by adding more distilled water and the reaction mixture in the flask was mixed thoroughly for homogenization. The absorbance was noted at 510 nm using a spectrophotometer. TFC were calculated using a standard calibration curve, prepared by analyzing catechin standard solutions within the concentration range of 10–100 mg/L (*R*^2^ = 0.9989). The amounts were reported as catechin equivalents (mg CE/100 g of dry matter). 

#### 3.5.5. DPPH.Scavenging Assay

2,2’-Diphenyl-1-picrylhydrazyl (DPPH) free radical scavenging capacity of the extracts was assessed following a previously described procedure [49]. In brief, freshly prepared DPPH methanolic solution (5.0 mL, 0.025 g/L) and the extract (1.0 mL, containing 25 μg/mL of dry matter in methanol) were mixed in a test tube. Absorbance of the reaction mixture was recorded at different time intervals (starting with 0 time to 10 min) at wavelength of 515 nm against a control. A control, containing all reagents, except the fruit extract was processed under similar conditions. Synthetic antioxidants, namely *tert*-butylhydroquinone (TBHQ), butylated hydroxyanisole (BHA) and propyl gallate (PG) at concentrations of 25 ppm were used as positive controls. The percent remaining amounts of DPPH radical (DPPH^.^) were calculated from the calibration curve. Absorbance measured at the 5th min was used for comparison of the radical scavenging activity of the extracts.

#### 3.5.6. Determination of Antioxidant Activity in Linoleic Acid System

The antioxidant activity of the tested apple fruit extracts was also determined by measuring the inhibition of linoleic acid peroxidation [50]. For this purposes, the extract (5 mg of each fruit) were added separately to an emulsion which contained solution of linoleic acid (0.13 mL), 99.8% ethanol (10 mL) and 10 mL of 0.2 *M* sodium phosphate buffer (pH 7). The mixture was made up to 25 mL with distilled water and incubated at 40 °C up to 360 h. The magnitude of linoleic acid oxidation was measured by peroxide data using the thiocyanate method as described by Yen *et al*. [51]. Briefly, in a flask, ethanol (10 mL, 75% v/v), aqueous solution of ammonium thiocyanate (0.2 mL, 30% w/v), sample solution (0.2 mL) and ferrous chloride (FeCl_2_) solution (0.2 mL, 20 mM in 3.5% HCl v/v) were added sequentially. After 3 min of vortexing, the absorbance of the reaction mixture was recorded at 500 nm with a spectrophotometer. A control, containing all reagents, except the extracts was also processed under similar conditions. Synthetic antioxidants, butylated hydroxytoluene (BHT) and butylated hydroxyanisole (BHA) were used as positive controls. Percent inhibition of linoleic acid oxidation was calculated using following equation: 

100 − [(Abs. increase of sample at 360 h/Abs. increase of control at 360 h) × 100]

#### 3.5.7. Determination of Reducing Power

The reducing power of the extracts was determined according to a previously described procedure [52] with slight modifications. The extracts (2.5–12.5 mg/mL) were mixed with sodium phosphate buffer (5.0 mL, 0.2 *M*, pH 6.6) and potassium ferricyanide (5.0 mL, 1.0%) in a test tube. The reaction mixture was incubated at 50 °C for 20 min in a water bath and then 10% trichloroacetic acid (5 mL) was added followed by centrifugation of the mixture at 980 g for 10 min at 5 °C (CHM-17; Kokusan Denki, Tokyo, Japan). After centrifugation, the upper layer of the solution (5.0 mL) was collected and diluted further by adding distilled water (5.0 mL) and ferric chloride (1 mL, 0.1%). The absorbance of the final solution was read at 700 nm using a spectrophotometer.

### 3.6. Mineral Composition

#### 3.6.1. Preparation of Samples for Mineral Analysis

Briefly, dried samples of apple peel and pulp (1.0 g in each case) were transferred into digestion flasks containing concentrated HNO_3_ (5 mL). Similarly, in another digestion flask, a blank sample was prepared by adding HNO_3_ (5 mL), but without the sample [53]. The flasks were heated for 2 h on an electric hot plate (HP 220, UTEC Products Inc., Albany, NY, USA) at 80 to 90 °C, after that, temperature was raised to 150 °C, letting to boiling the solution. Meanwhile, appropriate volume of concentrated HNO_3_ and 30% hydrogen peroxide (H_2_O_2_) were added to the flasks, occasionally and boiling continued until clear solutions were obtained indicating the completion of the digestion process and oxidation of the organic matter. The contents of the flasks were allowed to cool at room temperature then a small amount of distilled water was added and the solution filtered through Whatman No. 42 (<0.45-μm Millipore) filter paper. Finally, the volume was made up to 25-mL in a volumetric flask by adding distilled water.

#### 3.6.2. Preparation of Standards and Analysis of Samples

For quantitative purposes, working standard solutions of the elements namely sodium (Na), calcium (Ca), potassium (K), magnesium (Mg), iron (Fe), and zinc (Zn) were prepared from the stock standard solutions containing 1,000 mg/L of an element in 2 *N* nitric acid. Calibration and measurement of Ca, Mg, Fe and Zn was done on an atomic absorption spectrophotometer (A Analyst 300, Perkins Elmer, Waltham, MA, USA) whereas, Na and K were analyzed on a flame photometer (Sherwood, UK). Standard calibration curves were constructed for each element individually using linear correlation by least square method. A blank reading was also recorded to make necessary corrections during calculation of elemental concentrations.

### 3.7. Statistical Analysis

Three different samples of each fruit cultivar were randomly assayed. Each sample was analyzed individually in triplicate and data are reported as mean (*n* = 3× 3 × 1) ± SD (*n* = 9). The data generated was subjected to analysis of variance (ANOVA) using Minitab 2000 Version 13.2 statistical software (Minitab Inc., State College, PA, USA). The correlation analysis was done using the Pearson linear correlation method at the significance level of 0.05.

## 4. Conclusions

The phenolic components, antioxidant activity and mineral composition of peel and pulp tissues of different cultivars of apple fruit grown in Pakistan were compared. Nutritionally, among apple cultivars analyzed, the highest per serving basis contribution of total phenolics and total flavonoids was provided by Red Delicious, and the lowest by Sky Spur. On the other hand the Kala Kulu cultivar offered the maximum amount of minerals on a per serving basis than others. The peel of the apple cultivars tested exhibited higher contents of phenolics and better antioxidant activity coupled with higher amounts of valuable minerals compared to those in the pulp suggesting that fruit peel removal may induce more significant nutrient losses. As with many other fruits, vegetables and grains, apple consumption along with peel could provide more nutritive and medicinal benefits.

## References

[1] Abrosca B.D., Pacifico S., Cefarelli G., Mastellone C., Fiorentino A. (2007). Limoncella apple, an Italian apple cultivar: phenolic and flavonoid contents and antioxidant activity. Food Chem..

[2] Battino M., Beekwilder J., Denoyes-Rothan B., Laimer M., Mcdougall G.J. (2009). Bioactive compounds in berries relevant to human health. Nutr. Rev..

[3] Alberto M.R., Rinsdahl-Canavosio M.A., Manca de Nadra M.C. (2006). Antimicrobial effect of polyphenols from apple skins on human bacterial pathogens. Electron. J. Biotechnol..

[4] Wu J., Gao H., Zhao L., Liao X., Chen F., Wang Z., Hu X. (2007). Chemical compositional characterization of some apple cultivars. Food Chem..

[5] Wolfe K.L., Liu R.H. (2003). Apple peels as a value-added food ingredient. J. Agric. Food Chem..

[6] Boyer J., Liu R.H. (2004). Apple phytochemicals and their health benefits. Nutr. J..

[7] He H., Liu R.H. (2007). Triterpenoids isolated from apple peels have potent anti-proliferative activity and may be partially responsible for apple’s anticancer activity. J. Agric. Food Chem..

[8] Leontowicz M., Gorinstein S., Leontowicz H., Krzeminski R., Lojek A., Katrich E., Ciz M., Martin-Belloso O., Soliva-Fortuny R., Haruenkit R. (2003). Apple and pear peel and pulp and their influence on plasma lipids and antioxidant potentials in rats fed cholesterol-containing diets. J. Agric. Food Chem..

[9] Lata B. (2007). Relationship between apple peel and the whole fruit antioxidant content: Year and cultivar variation. J. Agric. Food Chem..

[10] Scalzo J., Politi A., Pellegrini N., Mezzetti B., Battino M. (2005). Plant genotype affects total antioxidant capacity and phenolic contents in fruit. Nutrition.

[11] Mangas J.J., Rodriguez R., Suarez B., Picinelli A., Dapena E. (1999). Study of the phenolic profile of cider apple cultivars at maturity by multivariate techniques. J. Agric. Food Chem..

[12] Podesedeic A., Wilska-Jeska J., Anders B., Markowski J. (2000). Compositional characterization of some apple varieties. Eur. Food Res. Technol..

[13] Gorinstein S., Zachwieja Z., Folta M., Barton H., Piotrowicz J., Zemser M., Weisz M., Trakhtenberg S., Martın-Belloso O. (2001). Comparative contents of dietary fiber, total phenolics, and minerals in persimmons and apples. J. Agric. Food Chem..

[14] Siddiqui B.N., Muhammad S., Malik N.H. (2006). Effect of socio-economic aspects on the awareness and adoption of recommended horticultural practices by apple growers in Baluchistan, Pakistan. Pak. J. Agric. Sci..

[15] Mukhtar A., Gilani H., Bhatty N. (2010). Some nutritional and microbiological aspects of apples of common varieties available for household consumption. J. Anim. Plant Sci..

[16] Muhammad A., Ayub M., Zeb A., Durrani Y., Ullah J., Afridi S.U.R. (2011). Physicochemical analysis of apple pulp from Mashaday variety during storage. Agric. Biol. J. N. Am..

[17] Abid M. (2005). Effect of Citric Acid with Lactic Acid on the Quality and Sensory Characteristics of Apple Drink. M.Sc. Thesis.

[18] (2006-2007). Agricultural Statistic of Pakistan 2006-2007.

[19] Vieira F.G.K., Borges G.D.S.C., Copetti C., Amboni R.D.D.M.C., Denardi F., Fett R. (2009). Physico-chemical and antioxidant properties of six apple cultivars (*Malus domestica* Borkh) grown in southern Brazil. Sci. Hortic..

[20] Drogoudi P.D., Michailidis Z., Pantelidis G. (2008). Peel and flesh antioxidant content and harvest quality characteristics of seven apple cultivars. Sci. Hortic..

[21] Henríquez C., Almonacid S., Chiffelle I., Valenzuela T., Araya M., Cabezas L., Simpson R., Speisky H. (2010). Determination of antioxidant capacity, total phenolic content and mineral composition of different fruit tissue of five apple cultivars grown in Chile. Chil. J. Agric. Res..

[22] Li Y., Guo C., Yang J., Wei J., Xu J., Cheng S. (2006). Evaluation of antioxidant properties of pomegranate peel extract in comparison with pomegranate pulp extract. Food Chem..

[23] Kuti J.O. (2004). Antioxidant compounds from four *Opuntia cactus* pear fruit varieties. Food Chem..

[24] Pan Y., He C., Wang H., Ji X., Wang K., Liu P. (2010). Antioxidant activity of microwave-assisted extract of *Buddleia officinalis* and its major active component. Food Chem..

[25] Wolfe K., Wu X., Liu R.H. (2003). Antioxidant activity of apple peels. J. Agric. Food Chem..

[26] Prasad K.N., Yang B., Zhao M., Sun J., Wei X., Jiang Y. (2010). Effects of high pressure or ultrasonic treatment on extraction yield and antioxidant activity of pericarp tissues of longan fruit. J. Food Biochem..

[27] Ozturk M., Ozturk F.A., Duru M.E., Topcu G. (2007). Antioxidant activity of stem and root extracts of Rhubarb (*Rheum ribes*): An edible medicinal plant. Food Chem..

[28] Chinnici F., Bendini A., Gaiani A., Riponi C. (2004). Radical scavenging activities of peels and pulps from cv. Golden Delicious apples as related to their phenolic composition. J. Agric. Food Chem..

[29] Sánchez-Moreno C., Larrauri J., Saura-Calixto F. (1999). Free radical scavenging capacity of selected red rosé and white wines. J. Sci. Food Agric..

[30] Cheng Z., Su L., Moore J., Zhou K., Luther M., Yin J.J., Yu L.L. (2006). Effect of postharvest treatment and heat stress on availability of wheat antioxidants. J. Agric. Food Chem..

[31] Stoilova I., Krastanov A., Bui H. (2008). Biodegradation of mixed phenolic compounds by a microbial association of *Aspergillus awamori* and *Thermoascus aurantiacus*. Electron. J. Environ. Agric. Food Chem..

[32] Durrani Y., Ayub M., Muhammad A., Ali A. (2010). Pysicochemical response of apple pulp to chemical preservatives and antioxidant during storage. Int.J. Food Saf..

[33] Ismail F., Anjum M.R., Mamon A.N., Kazi T.G. (2011). Trace metal contents of vegetables and fruits of Hyderabad retail market. Pak. J. Nutr..

[34] Soetan K.O., Olaiya C.O., Oyewole O.E. (2010). The importance of mineral elements for humans, domestic animals and plants: A review. Afr. J. Food Sci..

[35] Kumari M., Gupta S., Lakshmi A., Prakash J. (2004). Iron bioavailability in green leafy vegetables cooked in different utensils. Food Chem..

[36] Nour V., Trandafir I., Ionica M.E. (2010). Compositional characteristics of fruits of several apple (*Malus domestica*) cultivar. Not. Bot. Hort. Agrobot. Cluj.

[37] Ekholm P., Reinivuo H., Mattila P. (2007). Changes in the mineral and trace element contents of cereals, fruits and vegetables in Finland. J. Food Compost. Anal..

[38] Leterme P., Buldgen A., Estrada F., Londono A.M. (2006). Mineral content of tropical fruits and unconventional foods of the Andes and the rain forest of Colombia. Food Chem..

[39] Lin J.Y., Tang C.Y. (2007). Determination of total phenolic and flavonoid contents in selected fruits and vegetables, as well as their stimulatory effects on mouse splenocyte proliferation. Food Chem..

[40] Wijngaard H.H., Roble C., Brunton N. (2009). A survey of Irish fruit and vegetable waste and by-product as a source of polyphenolic antioxidants. Food Chem..

[41] Karadeniz F., Burdurlu H.S., Koca N., Soyer Y. (2005). Antioxidant activity of selected fruits and vegetables grown in Turkey. J. Agric. Food Chem..

[42] Mareezek A., Leja M., Ben J. (2000). Total phenolics, anthocyanins and antioxidant activity in the peel of the stored apples. J. Fruit Ornamental Plant Res..

[43] Silva E.M., Souza J.N.S., Rogez H., Rees J.F., Larondella Y. (2007). Antioxidant activities and polyphenolic contents of fifteen selected plant species from the Amazonian region. Food Chem..

[44] Sultana B., Anwar F., Przybylski R. (2007). Antioxidant activity of phenolic components present in barks of *Azadirachta indica*,* Terminalia arjuna*,* Acacia nilotica*, and *Eugenia jambolana* Lam. Trees. Food Chem..

[45] Sengul M., Yildiz H., Gungor N., Cetin B., Eser Z., Ercisli S. (2009). Total phenolic content, antioxidant and antimicrobial activities of some medicinal plants. Pak. J. Pharm. Sci..

[46] Association of Official Analytical Chemists (AOAC) (1990). Official Methods of Analysis of the Association of Official Analytical Chemists.

[47] Singleton V.L., Rossi J.A. (1965). Colorimetry of total phenolics with phosphomolybdic-phosphotungstic acid reagents. Am. J. Enol. Vitic..

[48] Zhishen J., Mengcheng T., Jianming W. (1999). The determination of flavonoid contents in mulberry and their screening effects on superoxide radicals. Food Chem..

[49] Brands-William W., Cuvelier M.E., Berset C. (1995). Use of a free radical method to evaluate antioxidant activity. Lebensm. Wiss. Technol..

[50] Osawa T., Namiki M. (1981). A novel type of antioxidant isolated from leaf wax of eucalyptus leaves. Agric. Biol. Chem..

[51] Yen G.C., Duh P.D., Chuang D.Y. (2000). Antioxidant activity of anthraquinones and anthrone. Food Chem..

[52] Oyaizu M. (1986). Studies on products of browning reaction prepared from glucosamine. Jpn. J. Nutr..

[53] Sahito A., Kazi T.G., Jakhrani M.A., Kazi G.H., Shar G.Q., Memon M.A. (2002). Elemental investigation of *Momordica charantia* Linn., and *Syziginm jambolana* Linn., using atomic absorption spectrophotometer. Nucleus.

